# *Culicoides* species composition and abundance on Irish cattle farms: implications for arboviral disease transmission

**DOI:** 10.1186/s13071-018-3010-6

**Published:** 2018-08-17

**Authors:** Áine B. Collins, John F. Mee, Michael L. Doherty, Damien J. Barrett, Marion E. England

**Affiliations:** 1 Animal and Bioscience Research Department, Teagasc, Moorepark, Fermoy, Co. Cork Ireland; 2 School of Veterinary Medicine, University College Dublin, Dublin 4, Ireland; 3 Surveillance, Animal By Products and TSE Division, Department of Agriculture, Food and the Marine, Backweston, Celbridge, Co. Kildare Ireland; 4 The Pirbright Institute, Ash Rd., Pirbright, Woking, Surrey GU24 0NF UK

**Keywords:** *Culicoides*, Ecological habitats, Schmallenberg virus, Bluetongue virus, Arbovirus, Vector, Sentinel herd surveillance, Ireland

## Abstract

**Background:**

Following the emergence of Schmallenberg virus (SBV) in Ireland in 2012, a sentinel herd surveillance program was established in the south of Ireland with the primary aim of investigating the species composition and abundance of *Culicoides* on livestock farms in the region.

**Methods:**

Ultraviolet-light trapping for *Culicoides* was carried out on 10 sentinel farms. Each site was sampled fortnightly over 16 weeks (21st July to 5th November 2014). One Onderstepoort Veterinary Institute UV light trap was run overnight at each site and catches were transferred immediately into 70% ethanol. *Culicoides* were morphologically identified to species level. Collection site habitats were characterised using the Phase 1 habitat survey technique (Joint Nature Conservation Committee).

**Results:**

A total of 23,929 individual *Culicoides* from 20 species was identified, including one species identified in Ireland for the first time, *Culicoides cameroni.* The most abundant species identified were *Culicoides obsoletus/Culicoides scoticus* (38%), *Culicoides dewulfi* (36%), *Culicoides pulicaris* (9%), *Culicoides chiopterus* (5%) and *Culicoides punctatus* (5%), comprising 93% of all *Culicoides* specimens identified. Collection site habitats were dominated by improved grassland and a combination of broadleaf woodland and native woodland species.

**Conclusions:**

The most abundant species of *Culicoides* identified were the putative vectors of bluetongue virus (BTV) and SBV in northern Europe. Their presence and abundance demonstrates the potential for future transmission of arboviruses among livestock in this region.

**Electronic supplementary material:**

The online version of this article (10.1186/s13071-018-3010-6) contains supplementary material, which is available to authorized users.

## Background

Arthropod-borne viruses (arboviruses) are transmitted by several insect vectors including mosquitoes and *Culicoides* biting midges [1]. *Culicoides* biting midges have been implicated in the transmission of over 50 arboviruses worldwide [1] including bluetongue virus (BTV; *Orbivirus*, *Reoviridae*), Schmallenberg virus (SBV; *Orthobunyavirus*, *Peribunyaviridae*) and African horse sickness virus (AHS; *Orbivirus*, *Reoviridae*). Currently, the only arbovirus known to be primarily transmitted by *Culicoides* to and between humans is Oropouche virus (OROV; *Orthobunyavirus*, *Peribunyaviridae*) [2]. The recent unprecedented emergence of arboviruses transmitted by *Culicoides* species in northern Europe, such as SBV and multiple serotypes of BTV, has highlighted Europe’s susceptibility to exotic arboviruses transmitted by biting midges from distant geographical regions.

Since 1998, there have been regular outbreaks of bluetongue disease in many parts of southern Europe with the Afro-Asiatic species, *Culicoides imicola*, implicated as the principal vector in the transmission of the virus [3]. However, BTV serotype 8 (BTV-8) emerged in northern Europe (the Netherlands) for the first time in 2006 [4]. The virus was successfully transmitted by northern Palaearctic species of *Culicoides*, specifically four members of the subgenus *Avaritia*; *Culicoides obsoletus* Meigen, 1818, *Culicoides scoticus* Downes & Kettle, 1952, *Culicoides dewulfi* Goetghebuer, 1936 and *Culicoides chiopterus* Meigen, 1830, and two members of the subgenus *Culicoides*; *Culicoides pulicaris* Linnaeus, 1758 and *Culicoides punctatus* Meigen, 1804 [5]. Subsequently, BTV was responsible for significant losses in livestock industries in a number of European countries between 2006 and 2008 [6]. Infection with BTV in ruminants can cause severe illness characterised by fever, inflammation of blood vessels (vasculitis), oedema and death in severe cases. More recently, BTV-8 re-emerged in France in 2015 [7] and outbreaks of bluetongue disease continue to occur in domestic livestock in France [8]. In October 2017, a consignment of cattle from France was imported into the UK, with some individuals testing PCR-positive for BTV [9]. *Culicoides* vector surveillance was immediately expanded to determine the risk of onwards transmission and monitoring of the situation is on-going (M. E. England, personal communication).

Schmallenberg virus is a novel Simbu serogroup *Orthobunyavirus* which emerged for the first time in northern Europe (Germany and the Netherlands) in 2011 [10]. The presence and abundance of suitable *Culicoides* vector species in northern Europe facilitated the rapid spread of SBV across the continent in 2012 resulting in a pan-European epizootic of Schmallenberg disease after a single vector-season [11]. The detection of SBV in field-caught *Culicoides* in a number of countries implicated a similar range of *Culicoides* species in the transmission of SBV as BTV [12–16]. Infection with SBV in ruminants can cause a drop in milk yield in dairy cattle, and abortions, stillbirths and congenital malformations in cattle, sheep and goats [10]. Following the initial European Schmallenberg epizootic in 2011/2012, the virus continued to circulate at a low level in a number of countries between 2013 and 2015. In contrast, there was little evidence of SBV circulation in Ireland in the three years (2013–2015) following the initial emergence of SBV in Ireland in 2012 [17, 18]. However, in 2016, SBV re-emerged and recirculated at a significant level in Ireland and the UK resulting in a second outbreak of congenital Schmallenberg disease in ruminants in late 2016 and early 2017 [19].

The emergence and re-emergence of BTV and SBV in Europe has highlighted the need for active surveillance systems for emerging and re-emerging infectious diseases. Arbovirus surveillance programs which combine serological, virological and vector studies are considered a particularly effective model for arbovirus surveillance [17]. A sentinel herd surveillance program (bovine serological and *Culicoides* entomological and virological studies) was, therefore, established on livestock farms located in the south of the Republic of Ireland (ROI) in order to monitor post-epizootic SBV circulation between 2013 and 2017 [17, 19].

A Department of Agriculture, Food and the Marine (DAFM) *Culicoides* survey conducted in Ireland between 2007 and 2009 as part of the National BTV Vector Surveillance Programme indicated the presence of several suspected *Culicoides* arbovirus vector species [20]. The most abundant species identified in this study were four members of the subgenus *Avaritia* (*C. obsoletus/C. scoticus C. dewulfi* and *C. chiopterus)* and two members of the subgenus *Culicoides* (*C. pulicaris* and *C. punctatus*) accounting for approximately 80–90% of all *Culicoides* identified. These species were found ubiquitously and in abundance throughout the ROI. The results of the DAFM study are consistent with similar studies in Northern Ireland and Scotland [21, 22]. Currently, a total of thirty *Culicoides* species are listed on the Irish *Culicoides* checklist [5]. However, limited data are available regarding the species and abundance of *Culicoides* biting midges in the south of Ireland. In the DAFM study, only two collection sites (one in Co. Kerry and one in Co. Waterford) out of a total of ten randomly selected sites distributed throughout the ROI were used to investigate the species composition of *Culicoides* in the same region as in the present study. Moreover, the DAFM study was conducted three years prior to the emergence of SBV in Ireland in 2012.

The south of Ireland is of particular interest compared to the rest of the country as it is where SBV first emerged in Ireland in 2012 [23] and re-emerged four years later in 2016 [19]. As a result, the south of Ireland is considered one of the most likely regions for BTV and other exotic arboviruses to enter Ireland. Therefore, detailed knowledge of the *Culicoides* composition in this region is essential to rapidly assess the risk of introduction and transmission of *Culicoides-*borne arboviruses such as BTV in Ireland. An in-depth *Culicoides* survey was established in ten sentinel farms in the south of Ireland in 2014 with the aim of investigating the species composition, abundance and broad ecological preferences of *Culicoides* biting midges in this region.

## Methods

### Collection sites

**Fig. 1:**
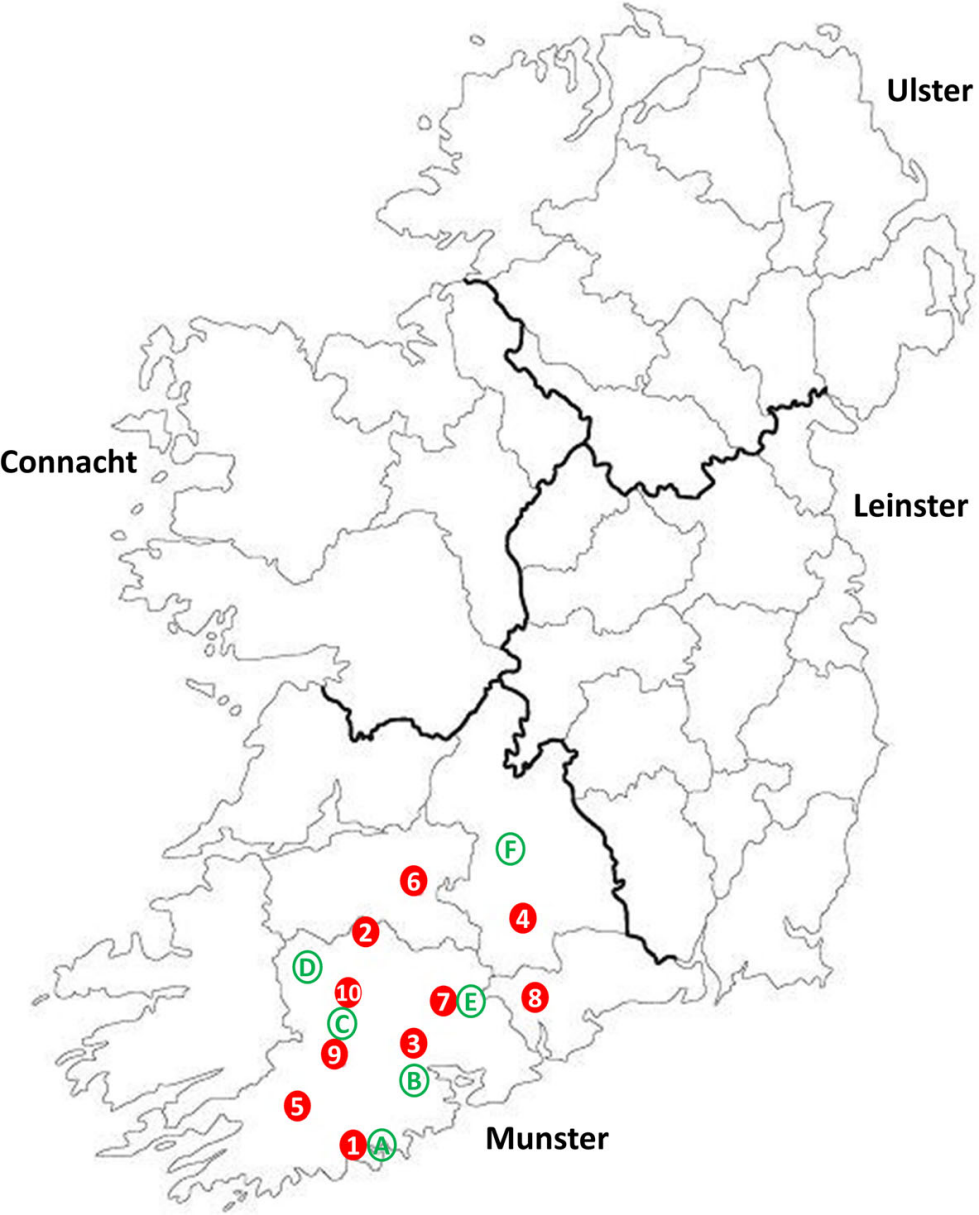
Map showing the location of the ten sentinel farms and six weather station locations in the south of the Republic of Ireland. Each numbered red dot (1–10) corresponds with each of the ten sentinel farms studied and each of the six alphabetically labelled green circles (A-F) corresponds with the location of the local weather stations. Seven farms were located in county Cork, the county where Schmallenberg virus was first identified in Ireland in 2012 [16], and one farm each was located in the adjoining counties Limerick, Tipperary and Waterford, respectively

As part of a Schmallenberg virus sentinel herd surveillance study, 26 livestock farms located in the south of Ireland were used to monitor post-epizootic SBV circulation in Ireland [17, 19]. Ten of these farms were selected, based on their geographical location to cover as great an area of the south of Ireland as possible, for collection and monitoring of *Culicoides* (Table 1 and Fig. 1).

**Table 1:** Characteristics of the ten sentinel farms located in the south of the Republic of Ireland included in the surveillance study for *Culicoides* in 2014 (July-November)

Farm ID	Location	Grid reference		Altitude (m)	Farm animals	No. of collections with *Culicoides*/total no. collections	Mean no. of *Culicoides* per collection	Maximum no. of *Culicoides* per collection
		Latitude	Longitude					
1	Clonakilty, Co. Cork	51.65°	-8.85°	76	230 Bovines; 100 Ovines^a^	5/8	52.2	121
2	Charleville, Co. Cork	52.33°	-8.79°	128	180 Bovines	7/8	593.3	2858
3	Carrignavar, Co. Cork	52.02°	-8.44°	177	212 Bovines	7/8	240.6	589
4	Cahir, Co. Tipperary	52.44°	-7.96°	45	390 Bovines	7/8	618.4	2216
5	Dunmanway, Co. Cork	51.71°	-9.19°	131	169 Bovines	6/8	269.2	874
6	Hospital, Co. Limerick	52.46°	-8.49°	79	241 Bovines	8/8	197.5	1265
7	Fermoy, Co. Cork	52.18°	-8.24°	37	324 Bovines; 26 Ovines^a^	7/8	327.1	1322
8	Tallow, Co Waterford	52.09°	-7.94°	70	677 Bovines	7/8	435.1	1282
9	Macroom, Co. Cork	51.88°	-9.01°	81	106 Bovines	6/8	353.0	975
10	Mallow, Co. Cork	52.13°	-8.76°	84	142 Bovines	8/8	375.9	1155
All	Cork (7); Waterford (1); Tipperary (1); Limerick (1)	51.65° to 52.44°	-9.19° to -7.94°	37–177	106–677 Bovines; 26–100 Ovines	68/80	354.2	2858

^a^Ovines grazed separately from bovines

### Specimen collection

Onderstepoort Veterinary Institute-type ultraviolet (UV) light suction traps were used to collect *Culicoides*; one trap was operated overnight (dusk until dawn) on each farm, approximately 250m away from livestock. Each farm was sampled fortnightly over a period of 16 weeks (21st July - 5th November 2014) during the 2014 vector season, corresponding to eight trap collections per farm and a total of 80 collections during the study period. Insects attracted to the UV light suction traps during operation were collected into beakers. Insects were transferred into 70% ethanol the following morning for storage pending specimen identification.

### Identification of specimens

Trap collections were identified morphologically to species-level under a dissecting microscope using the keys of Campbell & Pelham-Clinton [24] and Mathieu et al. [25]. Female *Culicoides* were further classified as unpigmented (nulliparous), pigmented (parous), gravid and blood-fed individuals. It is not possible to distinguish between *C. obsoletus* and *C. scoticus* females based on morphological characteristics, so they were grouped as *C. obsoletus/C. scoticus.* In contrast, it is possible to distinguish male *C. obsoletus* and *C. scoticus* specimens; hence, males of these species were morphologically identified and counted individually.

*Culicoides* specimens with damaged/missing abdomens (*n* = 478) were identified to species level but the parity status of those females was recorded as not-specified (N/S). Specimens that were too damaged to identify to species level (*n* = 75) were counted as unidentified *Culicoides.*

A number of specimens of *Culicoides clastrieri* Callot, Kremer & Deduit, 1962 were morphologically identified in trap catches suggesting a possible new *Culicoides* record for Ireland. However, considering that it can be difficult to separate *C. clastrieri* and *C. festivipennis* by wing morphology, molecular analyses were employed to explore the taxonomy of these two species. To do this, a region of the cytochrome *c* oxidase subunit 1 (*cox*1) gene of two female specimens morphologically identified as *C. clastrieri* from two different farms, were sequenced (adapted from Folmer et al. [26] and Hebert et al. [27]). These sequences were compared with available sequences in GenBank using BLAST [28].

### Habitat surveys

The habitat surrounding the trap at each of the ten locations was characterised using the Phase 1 habitat survey technique [29]. Each farm site was visited and the land use surrounding the trap (approximately 500 m) was categorised according to Phase 1 habitat survey classifications [29]. The relative density (low; +, medium; ++, high; ++) of habitat classes within the surveyed area was recorded. Target notes recorded the location of important on-farm features such as manure storage points (slurry pits, lagoons, dung heaps) and the location of the OVI trap. Altitude and the number of livestock on each farm were also noted.

### Meteorological data

Meteorological data (maximum, minimum and mean daily temperature in °C) were retrieved from the Irish Meteorological Service Online [30] and from Dr Patrick Touhy, Teagasc (personal communication) for six weather stations (A-F) located within the region (Fig. 1). One weather station each was located on Farm 1 (weather station A) and Farm 7 (weather station E), and four other weather stations (B, C, D and F) were located within a 25 km radius of one of the other study farms (Fig. 1). The correlation (Pearson’s correlation *r*) between mean fortnightly temperature at the six weather stations (mean of data from the 6 weather stations) and *Culicoides* abundance during the 16 week study period was calculated using GraphPad Prism 7 software (GraphPad Software Inc., CA, USA).

## Results

A total of 23,929 *Culicoides*, representing twenty species, was collected from 10 farms in 68 successful trap collections; twelve trap collections from 8 farms (range 1–3 catch collections per farm) yielded no *Culicoides* specimens (Tables 2, 3). *Culicoides* were found ubiquitously across all sites; however, there was large variation in the total number of *Culicoides* collected on each farm during the 16-week study period ranging from 257 to 4285 *Culicoides* per farm (Additional file 1: Figure S1). Female *Culicoides* (84%; *n* = 19,936) were more abundant than males (*n* = 3918; 16%) in trap catches, equating to a male to female sex ratio of 1:5.

**Table 2:** Species of *Culicoides*, sorted according to their abundance (number and percentage) and gender, on ten Irish farms collected in the south of the Republic of Ireland during part of the 2014 vector-active season (July-November)

Culicoides (*C.*) species	Female		Male			Total		No. of farms with species confirmed
	*n*	%	*n*	%		*n*	%	
*C. obsoletus/C. scoticus*	8103	89	1004^a^	11		9107	38	10
*C. dewulfi*	6755	78	1928	22		8683	36	10
*C. pulicaris*	1879	85	325	15		2204	9	10
*C. punctatus*	1056	94	67	6		1123	5	10
*C. chiopterus*	781	72	305	28		1086	5	10
Sub-total (vector species)	18,574	84	3629	16		22,203	93	
*C. achrayi*	1003	82	220	18		1223	5	10
*C. festivipennis*	175	83	35	17		210	< 1	9
*C. impunctatus*	88	88	12	12		100	< 1	8
*C. nubeculosus*	31	70	13	30		44	< 1	5
*C. circumscriptus*	35	90	4	10		39	< 1	7
*C. salinarius*	13	81	3	19		16	< 1	5
*C. fascipennis*	5	83	1	17		6	< 1	3
*C. delta*	3	75	1	25		4	< 1	3
*C. cameroni*	3	100	0	0		3	< 1	1
*C. brunnicans*	2	100	0	0		2	< 1	2
*C. newsteadi*	1	100	0	0		1	< 1	1
*C. riethi*	1	100	0	0		1	< 1	1
*C. stigma*	1	100	0	0		1	< 1	1
*C. reconditus*	1	100	0	0		1	< 1	1
Sub-total (other *Culicoides* spp.)	1362	82	289	18		1651	7	
Total	19,936	84	3918	16		23,854^b^	100	

^a^690 *Culicoides obsoletus* and 314 *Culicoides scoticus* males

^b^75 unidentifiable *Culicoides* (74 females and 1 male) not included in the table

**Table 3:** Current Irish *Culicoides* species list (*n* = 31) and species confirmed in the present study (*n* = 20), including one species recorded in Ireland for the first time

Genus	Species	Authority	Confirmed
*Culicoides* (*Avaritia*)	*chiopterus*	Meigen, 1830	✓
	*dewulfi*	Goetghebuer, 1936	✓
	*obsoletus*	Meigen, 1818	✓
	*scoticus*	Downes & Kettle, 1952	✓
*Culicoides* (*Beltranmyia*)	*circumscriptus*	Kieffer, 1918	✓
	*salinarius*	Kieffer, 1914	✓
*Culicoides* (*Culicoides*)	*delta*	Edwards, 1939	✓
	*grisescens*	Edwards, 1939	
	*impunctatus*	Goetghebuer, 1920	✓
	*newsteadi*	Austen, 1921	✓
	*pulicaris*	Linnaeus, 1758	✓
	*punctatus*	Meigen, 1804	✓
*Culicoides* (*Monoculicoides*)	*nubeculosus*	Meigen, 1830	✓
	*parroti*	Kieffer, 1922	
	*riethi*	Kieffer, 1914	✓
	*stigma*	Meigen, 1818	✓
*Culicoides* (*Oecacta*)			
*sensu* Szadziewski et al., 2016	*brunnicans*	Edwards, 1939	✓
	*vexans*	Campbell & Pelham-Clinton, 1960	
	*duddingstoni*	Kettle & Lawson, 1955	
	*festivipennis*	Kieffer, 1914	✓
	*heliophilus*	Edwards, 1921	
	*kibunensis*	Tokunaga, 1937	
	*pictipennis*	Staeger, 1839	
	*poperinghensis*	Callot, Kremer & Paradis, 1962	
Unspecified	*cameroni*	Staeger, 1839	✓^a^
	*furcillatus*	Goetghebuer, 1953	
	*reconditus*	Campbell & Pelham-Clinton, 1960	✓
	*segnis*	Campbell & Pelham-Clinton, 1960	
*Culicoides* (*Silvaticulicoides*)	*achrayi*	Kettle & Lawson, 1955	✓
	*fascipennis*	Staeger, 1839	✓
	*pallidicornis*	Kieffer, 1919	

^a^Species recorded for the first time in Ireland

**Fig. 2:**
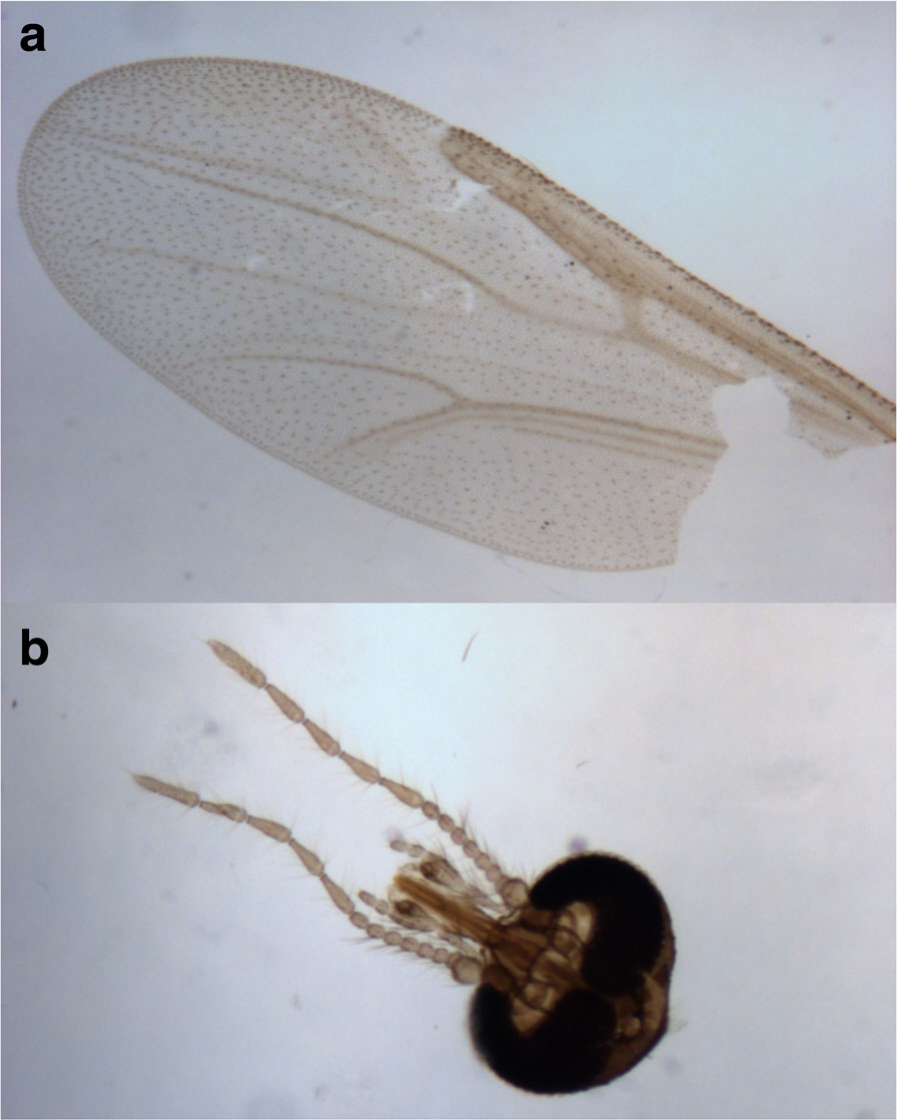
Photograph of wing (**a**) and head/antennae (**b**) of *Culicoides cameroni* identified in Ireland for the first time (photomicrograph taken at 10× magnification)

*Culicoides cameroni* Campbell & Pelham-Clinton, 1960, was identified and recorded in the ROI for the first time (Fig. 2). The most abundant species identified were members of the subgenus *Avaritia* (*C. obsoletus/C. scoticus*: 38%; *C. dewulfi*: 36%; and *C. chiopterus*: 5%), *C. pulicaris*: 9% and *C. punctatus*: 5%, comprising 93% of all *Culicoides* collected (Table 2 and Additional file 1: Figure S1). While *C. obsoletus* and *C. scoticus* females are indistinguishable morphologically, males were identified and counted separately; *C. obsoletus* males (*n* = 690) were more abundant than *C. scoticus* males (*n* = 314) (Table 2). The remaining *Culicoides* were principally *Culicoides achrayi* Kettle & Lawson, 1955 (5.1%) and *Culicoides festivipennis* Kieffer, 1914 (0.9%) (Table 2 and Additional file 1: Figure S1). The results of the molecular analyses on two specimens morphologically identified as *Culicoides clastrieri* Callot, Kremer & Deduit, 1962, revealed that both individuals had 99% match with both *C. festivipennis* and *C. clastrieri* and, therefore, we were unable to confirm *C. clastrieri* as a new record for ROI. Due to the apparent uncertainty surrounding the morphological and molecular distinction of these two species, *C. festivipennis* refers to individuals which were morphologically identified either as *C. festivipennis* or *C. clastrieri*. Seventy-five damaged *Culicoides* could not be identified to species level (74 females and 1 male). The overall species composition and relative abundance of the most frequently identified *Culicoides* species on the ten sentinel farms are illustrated in Additional file 1: Figure S1.

**Fig. 3:**
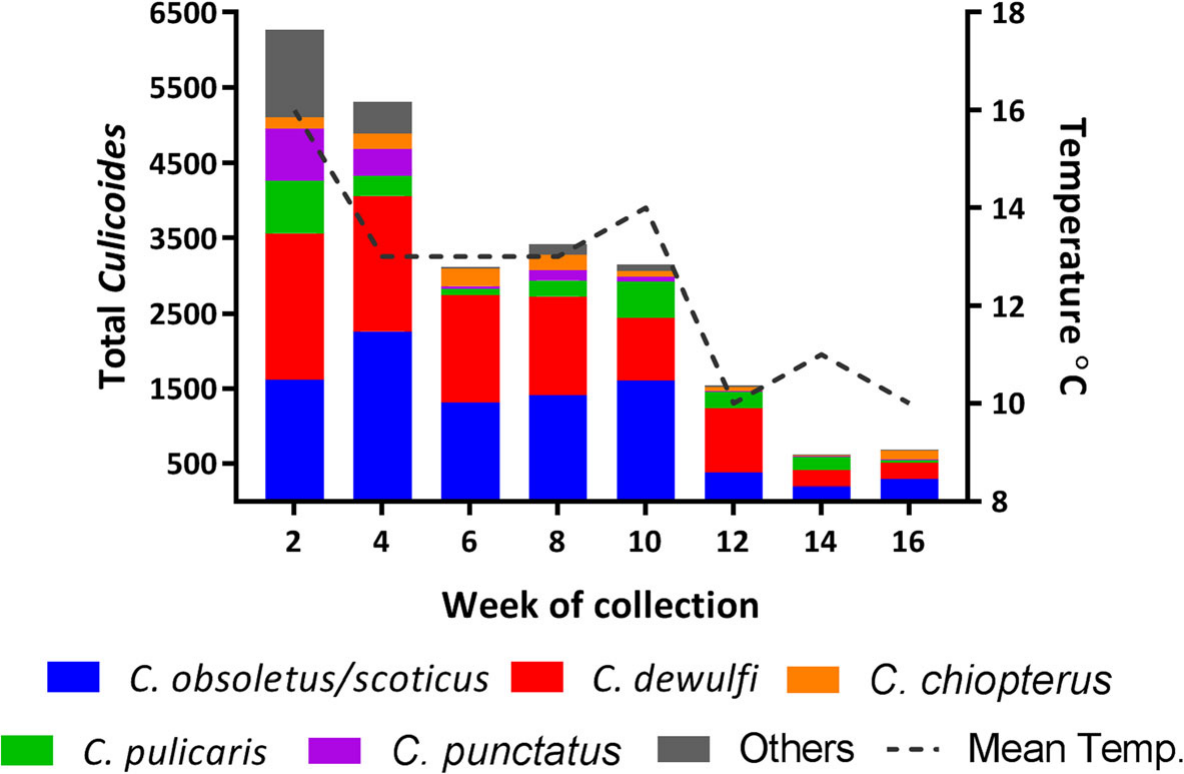
Total *Culicoides* abundance on 10 sentinel farms in the south of Ireland (between 21st July and 5th November 2014) in relation to week of collection and mean temperature at six weather stations located in the region

Mean fortnightly temperatures were highly correlated between the six weather stations (*r* range 0.90–0.99, *P* < 0.01). The abundance of *Culicoides* collected on the 10 farms during each fortnightly collection period was highly correlated with mean fortnightly temperature in the region (*r* = 0.87, *P* < 0.01) (Fig. 3). The abundance of *C. obsoletus/C. scoticus*, *C. dewulfi*, *C. pulicaris*, *C. punctatus* and other *Culicoides* species at each fortnightly collection were each significantly correlated with mean fortnightly temperatures in the region (*r* = 0.79, 0.77, 0.80, 0.77 and 0.76, respectively; all *P* < 0.05). In contrast, there was no significant correlation between mean fortnightly temperature and the relative abundance of *C. chiopterus* at each fortnightly collection (*r* = 0.46, *P* > 0.05). The majority (88%) of *Culicoides* were collected within the first 10 weeks (between 21st July and 28th September) of the study period (Fig. 3) which correlated with warmer temperatures.

The total number of *Culicoides* species identified ranged from 10 to 15 species per farm (mean = 13; Table 2) and this was weakly correlated (non-significantly) with total *Culicoides* abundance on each farm (*r* = 0.34, *P* > 0.05). There was no correlation between the number of host species per farm and the total number of *Culicoides* per farm (*r* = 0.17, *P* > 0.05). The six major *Culicoides* arbovirus vector species were identified on all 10 farms.

**Fig. 4:**
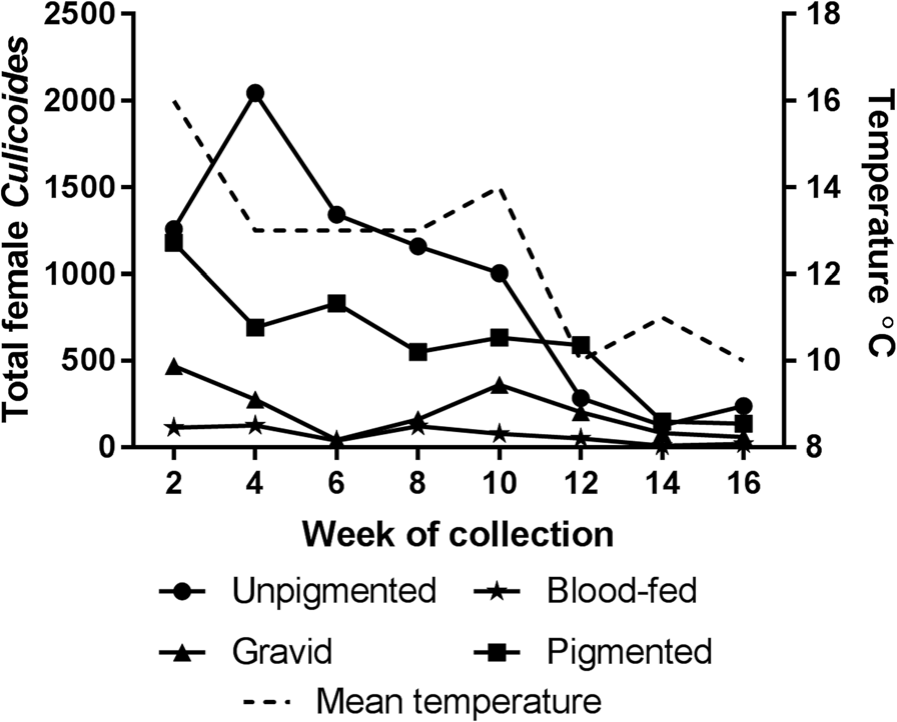
Change in *Culicoides* parity rate of *C. obsoletus/C. scoticus* and *C. dewulfi* and mean temperature across the collection period (between 21st July and 5th November 2014) on 10 sentinel farms located in the south of the Republic of Ireland

The parity status was determined for 98% (*n* = 19,458) of all female *Culicoides* collected (Additional file 2: Table S1). The majority of the female arbovirus vector species collected was unpigmented (46%) and pigmented (33%), followed by gravid (12%) and blood-fed (5%). For other *Culicoides* species, gravid *Culicoides* (33%) were the most abundant, followed by unpigmented (28%), pigmented (28%) and blood-fed (10%). The change in parity rate across the collection period is shown in Fig. 4. The overall abundance of each parity group (unpigmented, pigmented, gravid and blood-fed) of *Culicoides* were each significantly highly correlated with mean fortnightly temperatures (*r* range 0.74–0.83, *P* < 0.05).

Collection site habitat classes are summarised in Table 4. All 10 farms were dominated by improved grassland. Broadleaf woodland was present on most farms; coniferous woodland was also present at a lower density. Boundaries on farms were predominantly native woodland species. Non-native shrub/tree species boundaries were also present on farms to a lesser extent, typically around domestic buildings. The majority of farms had a water course running through the site and almost half of farms had a dense network of cow roadway systems. There were varying densities of broadleaf and coniferous scattered trees across the ten farms. On average, OVI-traps were stationed approximately 250 m (range 0–500 m) from livestock, approximately 120 m (range 20–155 m) from the main farmyard and approximately 130 m (range 20–155 m) from a manure storage point. Cattle were the most abundant host species present on study farms. Sheep were also present in fewer numbers.

**Table 4:** Farm and OVI-trap collection site habitat characteristics with total *Culicoides* abundance and catch break-down (%) per farm and relative density (low; +, medium; ++, high; +++) of habitat classes

Farm ID	1	2	3	4	5	6	7	8	9	10
Total no. *Culicoides*	257	4001	1687	4285	1651	1549	2257	3040	2105	3022
*C. obsoletus/C. scoticus* (%)	25	27	33	36	43	27	43	62	43	32
*C. dewulfi* (%)	32	35	51	42	30	55	24	17	44	39
*C. pulicaris* (%)	19	12	4	10	16	4	7	6	4	14
*C. punctatus* (%)	6	13	2	2	1	2	12	1	2	2
*C. chiopterus* (%)	12	2	5	8	1	10	6	1	6	3
Other *Culicoides* species	6	11	4	2	9	2	7	13	2	10
Animals (No.)										
Bovines	230	180	212	390	169	241	324	677	106	142
Ovines	100						26			
Altitude (m)	76	128	177	45	131	79	37	70	81	84
Grassland										
Improved	+++	+++	+++	+++	+++	+++	+++	+++	+++	+++
Semi-improved					++		+		+	+
Marshy					+					
Woodland/Scrub										
Broadleaf		++	++	++	++		+++	+	+++	+
Coniferous	+	+		++	+			++		
Scrub					+			+	++	+
Boundaries/Margins										
Native woodland species	+	+++	++	+++	+++	+	+	++	+++	+++
Non-native shrub/tree species	+	+	+	+	+	+			+	+
Stone walls	+++				++					
Scattered trees										
Broadleaf	+	+	+	++	+		++	+++	+	+
Coniferous			+	+	+					+
Cow roadways	+++	++	+	+	+	+++	+++	+++	+	++
Waterways/ditches										
Stream	P		P		P					
River				P		P	P		P	P
Dry ditch	P	P			P	P		P	P	P
OVI-trap distance (m) from										
Farmyard	100	150	100	100	10	150	500	20	25	20
Manure storage point	150	150	75	150	50	150	500	20	50	20

*Abbreviation*: P, present

## Discussion

The most abundant *Culicoides* species identified in the present study (*C. obsoletus/C. scoticus*, *C. chiopterus*, *C. dewulfi*, *C. pulicaris* and *C. punctatus*) are the putative vectors of BTV and SBV in northern Europe. Moreover, the majority (80%) of females identified within these species were host-seeking females (unpigmented and pigmented). The presence and dominance of putative arbovirus vectors demonstrates the potential for future transmission of BTV and SBV and other *Culicoides*-borne viruses among livestock in this region.

Nearly twice as many *C. obsoletus* male specimens were identified compared to *C. scoticus* male specimens suggesting that *C. obsoletus* was relatively more abundant. However, separation of *C. obsoletus* females from *C. scoticus* females would be required to fully assess the relative abundance of these two species. As females of these two species are indistinguishable morphologically, molecular assays would need to be employed to separate the species. Current methods for this are labour intensive as each *Culicoides* female has to be processed individually and, as such, males of these species are more commonly used as an indication of relative abundance.

*Culicoides obsoletus/C. scoticus* were the most abundant species identified in the present study. These species are known to breed in a wide variety of habitats including acid grassland, leaf litter, dung heaps and cow pats [31, 32]. These findings are consistent with the relatively high density of such habitats on farms in the present study. A similarly high abundance of *C. dewulfi* was also recorded on study farms. *Culicoides dewulfi* and *C. chiopterus* are particularly associated with high soil moisture and cattle manure [32, 33] and in this study, cattle were the most abundant livestock species present on these farms. *Culicoides pulicaris* and *C. punctatus* were present in lower numbers compared to *C. obsoletus/C. scoticus* and *C. dewulfi*. A previous study in Ireland indicated that *C. pulicaris* and *C. punctatus* tend to be more active earlier in the vector-active season (May) in Ireland [34] compared to the time when the present study was conducted (July-November).

In the present study, *Culicoides* abundance on the 10 farms was highly correlated with ambient temperatures in the region. This finding supports the previous results of McCarthy et al. [34] (Republic of Ireland), Jess et al. [22] (Northern Ireland) and Sanders et al. [21] (Scotland). Mean fortnightly temperatures were positively correlated with the relative abundance of *C. obsoletus/C. scoticus*, *C. dewulfi*, *C. pulicaris*, *C. punctatus* and other *Culicoides* species in the region but were not significantly correlated with relative abundance of *C. chiopterus.* This is most likely due to the fact that *C. chiopterus* was the least abundant species in the present study accounting for only 4.5% of all *Culicoides* trapped and identified. The small numbers of *C. chiopterus* may have influenced the outcome of the statistical analyses due to the small sample size. Some species such as *C. chiopterus* are frequently underrepresented in light traps, but further trapping could provide larger samples for analyses of these species.

In the present study, there was wide variation in total *Culicoides* abundance between farms. The greatest number of *Culicoides* was collected on Farm 2 and Farm 4, accounting for 17 and 18% of all *Culicoides* collected, respectively. In contrast, Farm 1 yielded the least number of *Culicoides* accounting for only 1% of all *Culicoides* collected. Ecological habitats, and possibly local meteorological conditions not captured in the present study such as wind speed and precipitation, may have influenced the *Culicoides* abundances on these farms. For example, Farm 1 was located close to the coast; this, coupled with the fact that this farm had a low density of woodland and trees, is likely to have resulted in the OVI-trap being more exposed to windy conditions, thus, resulting in smaller catch collections. Farm 2 and Farm 4 had noticeably higher densities of woodland and native woodland species in farm boundaries in comparison to Farm 1, which are likely to have provided shelter from the wind and suitable substrate (e.g. leaf litter) for larval development and emergence. Furthermore, the difference in distance between traps and animals (which can be quite variable and often not standardised in these types of studies) may have also been an important factor to explain differences in *Culicoides* abundances between farms in the present study.

The total *Culicoides* abundance between farms did not appear to be influenced by differences in host availability on individual farms. Total *Culicoides* abundance was not correlated with total number of cows and sheep per farm. The distance from trap to animal varied on each farm and may have contributed to differences in *Culicoides* abundance between farms. Additionally, it is difficult to be specific about host availability as all farms were commercial dairy herds with rotational grazing systems; the proximity of animals to the trap on each collection day may have varied during the study period as animals grazed different farm paddocks during each rotation. Therefore, the number of animals present on the farm can give a broad indication of host availability, but the animals may not always be in close proximity to the trap.

The total number of *Culicoides* species identified ranged between 10–15 species per farm. This was weakly correlated with total *Culicoides* abundance per farm, for example *Culicoides* species diversity was wide on Farm 10 (*n* = 15 *Culicoides* species; *n* = 4293 individuals) and narrow on Farm 1 (*n* = 10 *Culicoides* species; *n* = 264 individuals). The identification of *C. cameroni* in the present study constitutes a new Irish record. This new record in Ireland will update the current checklist of Irish *Culicoides* [5] to a total of 31 species. *Culicoides cameroni* is not considered an arbovirus vector species and has been recorded in the UK previously. There was some evidence to suggest that *C. clastrieri* was also present on farms in the present study (based on morphological identification); however, molecular analyses were unable to distinguish this species from *C. festivipennis* based on sequencing of the mitochondrial cytochrome *c* oxidase subunit 1 (*cox*1) gene. This has highlighted the need for further taxonomic investigation of these two species with specimens from a range of locations, as they appear to be almost indistinguishable based on the *cox*1 gene.

The parity dynamics of female *Culicoides* provides an indication of the changes in population age across the collection period. In the present study, the overall abundance of each parity group of *Culicoides* was significantly correlated with mean fortnightly temperatures demonstrating the influence of local meteorological conditions on the *Culicoides* life-cycle. There was a notable increase in the abundance of nulliparous *Culicoides* in the fourth week of the collection period (11th - 17th August). During the sixth week of collection (25th - 31st August), there was an increase in the abundance of pigmented *Culicoides* demonstrating the change in population age during the season*.*

Limited data are available regarding the species and abundance of *Culicoides* biting midges in the south of Ireland. In 2007, DAFM initiated the National BTV Vector Surveillance Program which collected *Culicoides* on a weekly basis at ten randomly selected sites located throughout the ROI between April 2007 and December 2009. Two sites were located in the south of Ireland; one in Co. Kerry and one in Co. Waterford [20]. Similar to the results of the present study, in the DAFM study the *Avaritia* and *Culicoides punctatus* and *Culicoides pulicaris* were the most abundant species identified (during the same 16-week study period as the present study) in 2007 (91%), 2008 (95%) and 2009 (81%).

However, it is interesting to note that the percentage composition of these species in the DAFM study differed considerably from the results of the present study, particularly in relation to the species *C. obsoletus/C. scoticus*, *C. chiopterus*, *C. pulicaris* and *C. punctatus*. In the present study *C. obsoletus/C. scoticus* accounted for 38% of all *Culicoides* identified. However, in the DAFM study the percentage composition of *C. obsoletus/C. scoticus* was lower in 2007 (18%), 2008 (14%) and 2009 (5%) during the same 16-week study period. The abundance of *C. chiopterus* was notably higher in the present study (5%) compared to the DAFM study in 2007 (0.9%), 2008 (0.3%) and 2009 (0.4%). The combined percentage of the *C. pulicaris* and *C. punctatus* in the present study (14%) was lower than the combined per cent for the same two species in the DAFM study in 2007 (37%), 2008 (20%) and 2009 (54%). While relative abundance of *C. dewulfi* in the present study (36%) was similar to the results of the DAFM study in 2007 (35%) and 2009 (22%), it was lower than that recorded in 2008 (60%). Given that the trapping methodology and insect traps used were the same in both studies, and the same 16 weeks of each year are compared directly here, it is interesting to note that during the five years between when the DAFM study was completed (2007–2009) and when the present study was completed (2014) the relative abundance of *C. obsoletus/C. scoticus* and *C. chiopterus* appears to have increased while the combined per cent of *C. pulicaris* and *C. punctatus* appears to have decreased*.* The population of each species may be affected differently by external factors such as predation, adverse weather conditions and changes in farming practices. As populations may take several years to recover following a decline, continued monitoring of *Culicoides* populations in the south of Ireland over a longer period of time coupled with data on changes in external factors affecting populations would be required to determine if this trend was continuing and the cause.

A number of studies have demonstrated that light-trapping surveillance does not always provide an accurate reflection of the biting population of *Culicoides* present. Frequently, certain individuals/species of *Culicoides* can be underrepresented in light-trap collection samples [35, 36]. In the present study, five-times more female *Culicoides* were identified compared to males despite the fact that male-to-female sex ratios in biting midge populations are assumed to be close to 1:1 [37]. While trapping techniques such as emergence traps typically show realistic sex ratios, skewed sex ratios in light traps have been reported in biting midges previously [32, 38]. Light traps are often female-biased with males regularly representing less than 5% of *Culicoide*s collected [35, 37, 39]. A number of hypotheses have been suggested to explain this phenomenon such as; light sources predominantly attract females more than males [40], males disperse over shorter distances from breeding sites compared to females [41] and females have a longer life span than males [42]. However, when considering disease risk, it is the female *Culicoides* that are most important and light traps provide an efficient and practical tool to investigate the faunal composition and abundance of *Culicoides* in an area. Indeed, ultraviolet light suction traps have been the most commonly used collection method in *Culicoides* surveys and national arbovirus surveillance programs since 2000 in southern Europe and since 2008 in northern and central Europe [35].

The possible underrepresentation of *C. chiopterus* should also be considered in the present study. The percentage composition of *C. chiopterus* in light-trap catches has been shown to be low in a number of studies previously, for example, in the ROI [34], in the UK [43], and in the Netherlands [44]. As a result, it has been assumed that *C. chiopterus* is likely to play a minor role in arbovirus transmission. However, Carpenter et al. [36] proposed that the role of *C. chiopterus* as an arbovirus vector may be markedly underestimated. A number of studies which collected *Culicoides* directly (e.g. drop trap, sticky tape trap, direct aspiration) from host species (bovine, ovine and equine) revealed high abundances of *C. chiopterus* in direct catch collections [36, 45, 46]. Hence, the low abundance of *C. chiopterus* in ultraviolet-light trap collections in the present study may not provide an accurate representation of this species in the surrounding area. Therefore, further studies that employ direct collection techniques may provide additional information about the species and abundance of *Culicoides* feeding on livestock in Ireland.

The effects of climate change and changes in meteorological conditions on the distribution and abundance of *Culicoides* and their ability to transport arboviruses into new regions [47] continues to pose a threat to livestock in many countries. Wind movement is considered an important factor in the transmission of exotic arboviruses from endemic regions into new regions. A model demonstrated that the majority of SBV infections in Ireland [48] and the UK [49] in 2012 occurred as a result of infected midges being transported through downwind movement facilitated by prevailing winds from continental Europe. A similar study implicated both downwind and upwind movements in the spatial and temporal pattern of BTV-8 movement across northwest Europe in 2006 [50]. It has also been proposed that the re-emergence of SBV in Ireland in 2016 was a result of favourable easterly wind conditions which may have facilitated the transport of virus-infected *Culicoides* into Ireland from neighbouring countries [19]. The on-going outbreak of BTV in France (2015-present) poses a continuing threat to livestock farms in Ireland and the UK, as has recently been the case [8, 9]. It is likely that, should BTV emerge in Ireland it will most likely occur as a result of wind dispersal of virus-infected *Culicoides* from neighbouring countries. A model has been developed by DAFM in conjunction with The Irish Meteorological Office (Met Éireann) to monitor weather conditions which may favour a possible incursion of *Culicoides* from the UK and continental Europe (D. J. Barrett, personal communication). The emergence of BTV or other exotic arboviruses in Ireland could result in dramatic disease epizootics due to the immunologically naïve status of animals in the region.

While virus-infected insects are the most likely route of entry of exotic arboviruses into Ireland, the possibility of importing virus-infected animals should also be considered. Bluetongue virus serotype 8 emerged on a cattle farm in Northern Ireland during the 2008 vector-free period following the importation of pregnant cattle from the Netherlands. All 20 cows tested negative for viral RNA (reverse transcription PCR) at importation, but three calves from two cows tested positive for BTV at birth [51]. Fortunately, this incident was rapidly isolated and there was no further transmission beyond the original herd. Between 2011 and 2016, on average, 6470 live animals (range: 2497–12,996) were imported into the ROI annually (excluding livestock imported for immediate slaughter), the majority of which (mean = 78%, range: 67–91% across the six years) were imported from the UK [52]. The remainder of livestock imports originated primarily in Denmark (4.7%), Germany (4.6%), France (3.8%) and the Netherlands (3.7%) [52]. It would be prudent to continue monitoring livestock imports as a possible route of introduction of exotic arboviruses into Ireland. In the context of BTV, SBV and other exotic arboviruses, continued monitoring of the dynamics of *Culicoides* biting midges on farms in Ireland is recommended, particularly considering the apparent change in the *Culicoides* species composition and abundance in the south of Ireland since 2009 and the on-going threat of a possible incursion of BTV-infected *Culicoides* from Europe. Evaluating *Culicoides* abundances throughout the year would also provide valuable information on adult *Culicoides* activity; knowing when the adult midges are active (and inactive) would indicate when disease transmission is most likely to occur. This information can be used by policy makers to inform decisions regarding animal movement restrictions and international trade.

## Conclusions

The most abundant *Culicoides* species identified in this study are the putative vectors of a number of arboviruses in northern Europe. The presence and abundance of these species highlight that disease transmission could occur and be maintained following a new incursion of BTV, SBV or other exotic *Culicoides*-transmitted arboviruses into these areas.

## Additional files

Additional file 1: **Figure S1.** Between-site variations in total *Culicoides* abundance on 10 sentinel farms (Farms 1–10) in the south of the Republic of Ireland. (TIF 250 kb)

Additional file 2. **Table S1.** Physiological/parity status of female *Culicoides* with the potential to transmit arboviruses collected on 10 Irish farms during part of the 2014 vector-active season (July-November). (DOCX 19 kb)

## Abbreviations

AHS: African horse sickness virus
BTV: bluetongue virus
Co.: County
*cox*1: cytochrome *c* oxidase subunit 1
OROV: Oropouche virus
OVI: Onderstepoort Veterinary Institute
ROI: Republic of Ireland
SBV: Schmallenberg virus
UV: ultraviolet
