# Supplementary figures and images for: Culicoides species composition and abundance on Irish cattle farms: implications for arboviral disease transmission
Source: Parasit Vectors. 2018 Aug 17;11:472. doi: 10.1186/s13071-018-3010-6 (PMC6098625; doi:10.1186/s13071-018-3010-6)

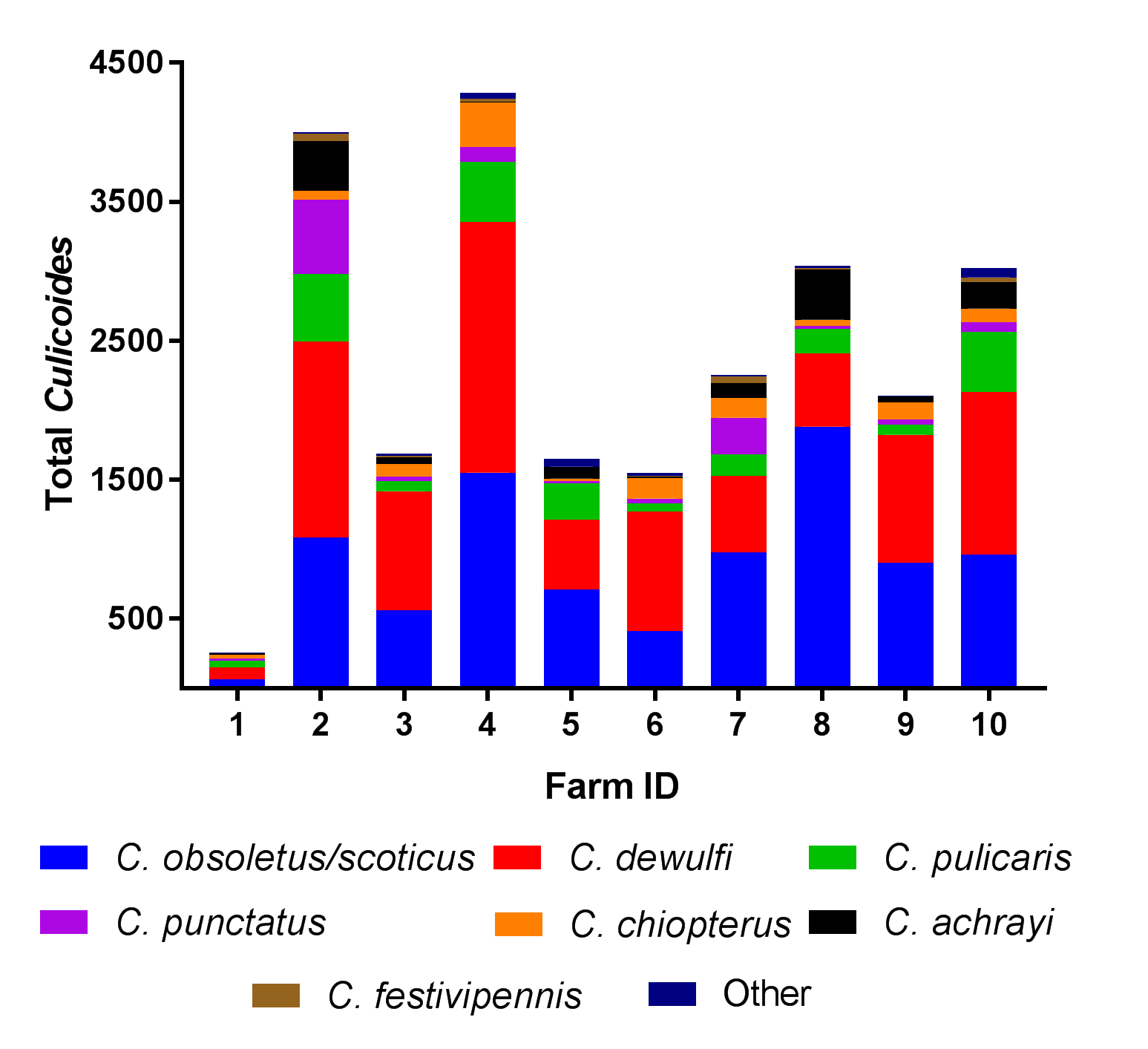

Supplement: Supplementary file 1 — Figure S1. Between-site variations in total Culicoides abundance on 10 sentinel farms (Farms 1–10) in the south of the Republic of Ireland. (TIF 250 kb) [file 13071_2018_3010_MOESM1_ESM.tif]
