# Supplementary material for: Culicoides species composition and abundance on Irish cattle farms: implications for arboviral disease transmission
Source: Parasit Vectors. 2018 Aug 17;11:472. doi: 10.1186/s13071-018-3010-6 (PMC6098625; doi:10.1186/s13071-018-3010-6)
Supplement: Supplementary file 2 — Table S1. Physiological/parity status of female Culicoides with the potential to transmit arboviruses collected on 10 Irish farms during part of the 2014 vector-active season (July-November). (DOCX 19 kb) [file 13071_2018_3010_MOESM2_ESM.docx]

| **Additional file 2.** Physiological/parity status of female *Culicoides* with the potential to transmit arboviruses collected on 10 Irish farms during part of the 2014 vector-active season (July-November). | | | | | | | | | | | | | | | | | |
| --- | --- | --- | --- | --- | --- | --- | --- | --- | --- | --- | --- | --- | --- | --- | --- | --- | --- |
| Culicoides (C.) Species | Unpigmented | |  | Pigmented | |  | Blood-fed | |  | Gravid | |  | N/S^a^ | |  | Total | |
|  | No. | % |  | No. | % |  | No. | % |  | No. | % |  | No. | % |  | No. | % |
| *C. obsoletus/C. scoticus* | 4112 | 51 |  | 2364 | 29 |  | 245 | 3 |  | 1001 | 12 |  | 381 | 5 |  | 8103 | 41 |
| *C. dewulfi* | 3350 | 50 |  | 2402 | 36 |  | 314 | 5 |  | 656 | 10 |  | 33 | 0 |  | 6755 | 34 |
| *C. pulicaris* | 746 | 40 |  | 767 | 41 |  | 292 | 16 |  | 39 | 2 |  | 35 | 2 |  | 1879 | 9 |
| *C. punctatus* | 168 | 16 |  | 356 | 34 |  | 97 | 9 |  | 419 | 40 |  | 16 | 2 |  | 1056 | 5 |
| *C. chiopterus* | 226 | 29 |  | 332 | 43 |  | 20 | 3 |  | 201 | 26 |  | 2 | 0 |  | 781 | 4 |
| Sub-total (arbovirus vector species) | 8602 | 46 |  | 6221 | 33 |  | 968 | 5 |  | 2316 | 12 |  | 467 | 3 |  | 18,574 | 93 |
| *C. achrayi* | 325 | 32 |  | 294 | 29 |  | 127 | 13 |  | 251 | 25 |  | 6 | 1 |  | 1003 | 5 |
| *C. festivipennis* | 41 | 25 |  | 26 | 16 |  | 8 | 5 |  | 86 | 53 |  | 2 | 1 |  | 163 | <1 |
| *C. impunctatus* | 6 | 7 |  | 45 | 51 |  | 0 | 0 |  | 36 | 41 |  | 1 | 1 |  | 88 | <1 |
| *C. nubeculosis* | 2 | 6 |  | 6 | 19 |  | 4 | 13 |  | 18 | 58 |  | 1 | 3 |  | 31 | <1 |
| *C. circumscriptus* | 0 | 0 |  | 3 | 9 |  | 0 | 0 |  | 32 | 91 |  | 0 | 0 |  | 35 | <1 |
| *C. clastrieri* | 4 | 33 |  | 1 | 8 |  | 2 | 17 |  | 5 | 42 |  | 0 | 0 |  | 12 | <1 |
| *C. salinaruis* | 1 | 8 |  | 0 | 0 |  | 0 | 0 |  | 12 | 92 |  | 0 | 0 |  | 13 | <1 |
| *C. fascipennis* | 1 | 20 |  | 1 | 20 |  | 1 | 20 |  | 2 | 40 |  | 0 | 0 |  | 5 | <1 |
| *C. delta* | 0 | 0 |  | 2 | 67 |  | 0 | 0 |  | 1 | 33 |  | 0 | 0 |  | 3 | <1 |
| *C. cameroni* | 0 | 0 |  | 2 | 67 |  | 0 | 0 |  | 0 | 0 |  | 1 | 33 |  | 3 | <1 |
| *C. brunnicans* | 0 | 0 |  | 0 | 0 |  | 0 | 0 |  | 2 | 100 |  | 0 | 0 |  | 2 | <1 |
| *C. newsteadi* | 0 | 0 |  | 0 | 0 |  | 0 | 0 |  | 1 | 100 |  | 0 | 0 |  | 1 | <1 |
| *C. riethi* | 1 | 100 |  | 0 | 0 |  | 0 | 0 |  | 0 | 0 |  | 0 | 0 |  | 1 | <1 |
| *C. stigma* | 0 | 0 |  | 0 | 0 |  | 0 | 0 |  | 1 | 100 |  | 0 | 0 |  | 1 | <1 |
| *C. reconditus* | 1 | 100 |  | 0 | 0 |  | 0 | 0 |  | 0 | 0 |  | 0 | 0 |  | 1 | <1 |
| Sub-total (Other *Culicoides* species) | 382 | 28 |  | 380 | 28 |  | 142 | 10 |  | 447 | 33 |  | 11 | 1 |  | 1362 | 7 |
| *Total* | 8984 | 45 |  | 6601 | 33 |  | 1110 | 6 |  | 2763 | 14 |  | 478 | 2 |  | 19,936^b^ | 100 |
| ^a^ Physiological status not-specified (N/S) | | | | | | | | | | | | | | | | | |
| ^b^ 74 unidentified female *Culicoides* not included in the table | | | | | | | | | | | | | | | | | |
